# Translation Stress Regulates Ribosome Synthesis and Cell Proliferation

**DOI:** 10.3390/ijms19123757

**Published:** 2018-11-27

**Authors:** Sivakumar Vadivel Gnanasundram, Robin Fåhraeus

**Affiliations:** 1 Inserm UMRS1162, Institut de Génétique Moléculaire, Université Paris 7, Hôpital St. Louis, F-75010 Paris, France; robin.fahraeus@inserm.fr; 2 RECAMO, Masaryk Memorial Cancer Institute, Zluty kopec 7, 65653 Brno, Czech Republic; 3 Department of Medical Biosciences, Building 6M, Umeå University, 901 85 Umeå, Sweden; 4 ICCVS, University of Gdańsk, Science, ul. Wita Stwosza 63, 80-308 Gdańsk, Poland

**Keywords:** mRNA translation stress, ribosome biogenesis, oncogene, cell cycle, cell signaling pathway

## Abstract

Ribosome and protein synthesis are major metabolic events that control cellular growth and proliferation. Impairment in ribosome biogenesis pathways and mRNA translation is associated with pathologies such as cancer and developmental disorders. Processes that control global protein synthesis are tightly regulated at different levels by numerous factors and linked with multiple cellular signaling pathways. Several of these merge on the growth promoting factor c-Myc, which induces ribosome biogenesis by stimulating Pol I, Pol II, and Pol III transcription. However, how cells sense and respond to mRNA translation stress is not well understood. It was more recently shown that mRNA translation stress activates c-Myc, through a specific induction of E2F1 synthesis via a PI3Kδ-dependent pathway. This review focuses on how this novel feedback pathway stimulates cellular growth and proliferation pathways to synchronize protein synthesis with ribosome biogenesis. It also describes for the first time the oncogenic activity of the mRNA, and not the encoded protein.

## 1. Human Ribosome Biogenesis—Overview

The ribosome is a complex macromolecule which constitutes the catalytic platform for mRNA translation [1]. Recent advancements in cryo-electron microscopy (cryo-EM) and high-resolution atomic crystal structures have provided a deeper understanding of the ribosomal assembly process and function, while also revealing how ribosomal RNAs (rRNAs) constitute a core part of the ribosome along with the ribosomal proteins embedded on the surface [2,3,4,5,6,7,8,9,10,11]. The core structure of the ribosome is conserved across all living cells [1,3]. Human ribosomes consist of two basic subunits, with variable compositions of ribosomal proteins and expansion segments in each subunit compared to other living cells. A small subunit (40S) consists of 18S rRNA and 33 small ribosomal proteins (RPSs) and a large subunit (60S) consists of 28S, 5S, and 5.8S rRNAs and 47 large ribosomal proteins (RPLs) [12,13,14]. The small subunit (40S) is the t-RNA binding, decoding, and mRNA passage site whereas the large subunit (60S) provides a GTPase binding platform, peptidyl transfer, and polypeptide exit tunnel [3,15,16].

Ribosome biogenesis (RiBi) is a dynamic and complex metabolic process which occurs in all living cells and reflects the necessity to control the synthesis of individual proteins and to coordinate cellular growth with cell proliferation. Human ribosome biogenesis was believed to be conserved across its mammalian counterparts. However, recent studies suggested that this process is more complex in human cells [17,18,19,20,21]. RiBi initiates with the synthesis of the 47S pre-rRNA (precursor for 18S, 5.8S, and 28S rRNAs) from RNA polymerase I (Pol I) promoter in the nucleolus and involves the participation of at least three prime factors; the ribosomal DNA transcription factor RRN3, Upstream Binding Factor (UBF), and Selectivity Factor 1 (SL1). This is a key rate-limiting step of the RiBi process at the Pol I transcriptional level and serves as a key target in cancer cells [22,23,24,25,26]. The transcribed pre-rRNAs are assembled with ribosomal proteins, undergo various site-specific base modifications (methylation, acetylation, and pseudouridylation) and ribose methylation (2′-O-methylation), and are then processed through a series of cleavages (via both exo- and endonucleases) to generate the functional ribosomes in the cytoplasm, where the final stages of maturation take place. The 5S rRNA which forms part of the large subunit core is transcribed separately from Pol III promoter in the nucleoplasm [14,27,28]. To ensure the fidelity, this complex process is regulated spatio-temporally at multiple steps orchestrated by numerous trans-acting factors, including ribonucleoproteins (RNPs), and enzymatic proteins [17] (see reviews [9,14,27,29]).

## 2. Ribosome Biogenesis Nexus with Cell Proliferation

**Figure 1:**
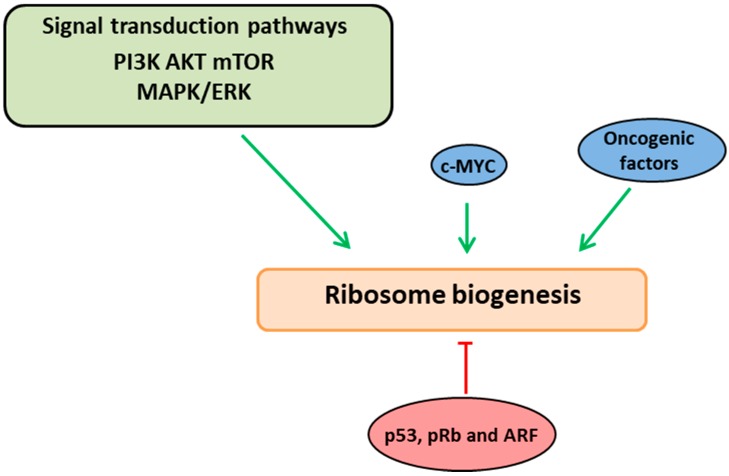
Ribosome biogenesis links with growth and cell proliferation pathways. Ribosome biogenesis is interlinked and coordinated with the cell cycle phases through a series of signaling cascades (PI3K-AKT-mTOR and ERK/MAPK pathways). These pathways induce ribosome biogenesis at various stages by acting directly on RNA polymerases or via the oncogenic factor, c-Myc (indicated in green arrows). Altered ribosome biogenesis is sensed by quality control check points which activate/stabilize the tumor-suppressor proteins (p53, ARF and pRb) resulting in negative regulation of ribosome biogenesis (indicated in red arrow).

Therefore, RiBi is critical for providing the protein levels required for cellular viability and proliferation and it is not surprising that in actively dividing cells, the rate of RiBi is tightly coordinated with cell cycle phases through a series of cell signaling pathways [24,30,31,32]. Mitogens and extracellular growth factors can induce the growth proliferation pathways such as PI3K/AKT and MAPK/ERK, which indeed trigger the RiBi by activating the Pol I through the phosphorylation of UBF and activate Pol III transcription by phosphorylating TFIIIB [24,28,33,34]. Another major pathway which positively regulates ribosome biogenesis is the mammalian target of the rapamycin (mTOR) pathway, which is composed of two major protein complexes, mTORC1 and mTORC2 [13,35,36,37]. Upon the stimulus from the growth factors and oncogenic signaling, mTORC1 induces RiBi at various stages, primarily it increases the Pol I transcription by activating UBF and TIF-IA and increases the Pol III transcription by associating the factors TFIIIB and TFIIIC with 5S rRNA genes [13,38,39,40]. Furthermore, mTORC1 increases the RiBi by actively promoting the increased translation of ribosomal proteins through its 5′ terminal oligo-pyrimidine (TOP) motifs. Although mTORC1 was known to regulate RiBi in most of the cases rather than mTORC2, it was reported that mTORC2 interacts with mature ribosomes and promotes the AKT survival pathway in proliferating cells [13,41] (see reviews [13,40]). Most of these pathways activate c-Myc which is a major regulatory factor of ribosome assembly by acting on all three RNA polymerases. Myc activates Pol I transcription by recruiting the factor SL1 to rDNA promoters and Pol III transcription by activating TFIIIB. It also induces Pol II transcription, resulting in the increased synthesis of ribosomal proteins, small nucleolar ribonucleoproteins (snoRNPs), and other assembly factors, see Figure 1 [24,42,43,44].

Pol I transcription is suppressed during mitosis by Cyclin-dependent kinase 1 (CDK1)-cyclin B kinase activity and can be reactivated following the inhibition of this kinase activity [25,45,46,47,48]. Another RiBi checkpoint factor is retinoblastoma protein (pRb), which, in its normal active form, binds to factors UBF and TFIIIB and inhibits the rRNA synthesis [25,49,50,51,52,53,54]. pRb also sequesters E2F transcription factors, which control the expression of the set of genes responsible for the promotion of the S phase in the cell cycle [55,56,57]. During the cell cycle progression, pRb is phosphorylated by the cyclin-D-CDK-4, cyclin-D-CDK-6, and cyclin-E-CDK-2 kinases resulting in the hindrance of pRb binding to UBF and TFIIIB, thereby facilitating the Pol I and Pol III transcription [25]. Phosphorylation of pRb also releases E2F which then promotes the S phase of the cell cycle. The interaction between pRb–E2F serves as the target for various oncogenic viruses such as the human papillomavirus (HPV), Simian virus 40 (SV40), and adenovirus that act via E7, Large T, and E1A, respectively, to compete with E2F binding to pRb [55,58,59,60].

Phosphorylation of pRb is indirectly regulated by the tumor-suppressor protein, p53, which upon activation increases the transcription of p21, thereby suppressing the CDK activity required for the phosphorylation of pRb [25,61,62]. It has been suggested that alteration or impairment in the RiBi process can release free ribosomal proteins, such asRPL5 and RPL11, that can modulate the levels of p53 and induce cell cycle arrest by preventing Mouse double minute 2 homolog (MDM2)-mediated degradation of p53 [24,63,64,65,66,67,68].

## 3. Ribosomopathies and Cancer

Impairment in the ribosome biogenesis results in the generation of dysfunctional ribosomes which are associated with collective disorders called ribosomopathies. These include Diamond-Blackfan Anemia (DBA), 5q− syndrome, Schwachman-Diamond syndrome (SDS), Treacher Collins syndrome (TCS), an X-linked form of dyskeratosis congenita, and cartilage-hair hypoplasia [69,70,71,72]. These are rare diseases that are often due to mutations or deletions in the encoding genes of ribosomal proteins or the biogenesis factors involved in this process [73,74], see review [75]. Despite the common nature of defects, the resulting phenotypes of these disorders are often heterogeneous. Interestingly, why the impaired ribosome biogenesis selectively affects certain cell types but not others remains elusive. Multiple hypotheses were proposed to explain this scenario; (i) Variability in the activation of p53 response pathways in mediating the ribosomopathies resulting in differential cellular phenotypes [76]. (ii) Another hypothesis is based on a ribosome concentration model; impaired ribosome synthesis reduces the global translation in all cells but due to variation in the translation initiation rate of certain mRNAs and depending on the cell’s overall reliability on translation, certain cell types are more sensitized compared to others [77]. (iii) Intriguingly, another hypothesis suggests that a heterogeneous composition of ribosomes (called ‘specialized ribosomes’) is present in different tissues/cell types favoring the translation of particular subsets of mRNAs based on different conditions/stress [78,79,80,81]. Overall, these studies indicate that differences in the cellular response towards ribosomopathies could be influenced by multiple parameters, such as ribosomal surveillance mechanisms and rescue, variation in mRNA translation rate, and compensatory allelic mutations, resulting in the restoration of the ribosome biogenesis process [77]. 

Numerous studies have shown a strong correlation between the deregulated ribosome biogenesis and cancer. However, the precise molecular mechanism causing this deregulation remains elusive. Interestingly, recent findings suggest that both quantitative, as well as qualitative, modifications in ribosomes are attributed to tumor progression [12]. In the case of a quantitative change in RiBi, the principal target could ideally be the Pol I complex as it is involved in the prime rate-limiting step of RiBi [28,82]. The terminal points of signal transduction pathways such as Myc, mTOR, and ERK stimulate the Pol I activity. These endpoints are often deregulated during tumor genesis, resulting in an increased rate of the RiBi. Additionally, the functional loss of tumor-suppressor proteins such as p53, ARF, pRb, and PTEN results in the deregulated Pol I transcription which is attributed to increased RiBi. Increased Pol I activity causes the upregulation of pre-rRNA synthesis, resulting in hyperactive RiBi, this leads to an increase in the global translation rate and altered pattern of mRNA translation, thereby contributing to the tumor growth [12,28,82]. In line with this, the first experimental evidence of hyperactive ribosome biogenesis contributing to tumor progression was reported in an Eµ-Myc-driven transgenic mouse model of Burkitt lymphoma. The overexpression of Myc triggers increased RiBi leading to lymphomagenesis. When Eµ-Myc/+ transgenic mice were crossed with RPL24^+/−^ and RPL38^+/−^ mice, RiBi was restored back to normal and tumor progression was reduced [83]. Following this, several studies were reported about the correlation of hyperactive RiBi with tumor initiation and progression [24,84,85,86]. The molecular mechanism of how the increased RiBi leads to cancer remains elusive. A possible explanation for this could be the malfunctioning of increased ribosome biogenesis checkpoints (IRBC) due to altered levels/functional loss of tumor-suppressor proteins [12].

Qualitative modifications in ribosomes occur mainly due to missense mutations or deletions in genes encoding the ribosomal proteins and alteration in the post-transcriptional modifications of rRNAs such as 2′-O-ribose methylation or pseudouridylation. 2′-O-ribose methylation was enhanced by either functional loss of p53 or via the activation of Myc, resulting in the increased activity of rRNA 2′ O methyltransferase fibrillarin and subsequent increase in the IRES-mediated translation of oncogenic mRNAs in addition to causing translation infidelity leading to tumor progression [12,87,88,89,90,91]. Another rRNA base modification, pseudouridylation, is mediated by the enzyme pseudouridine synthase dyskerin encoded by the *DKC1* gene. Mutations in the *DKC1* gene are largely reported with X-linked dyskeratosis congenita, resulting in decreased levels of rRNA pseudouridylation leading to decreased IRES-mediated translation of the subset of tumor-suppressor-encoding mRNAs and anti-apoptotic proteins. This also causes translation infidelity by impairing tRNA binding [92,93,94]. Overall, these qualitative alterations during RiBi are reported to generate ribosome heterogeneity, which could, in turn, affect the translation fidelity and augment the rate of internal ribosome entry site (IRES)-dependent translation of a subset of mRNAs and, thereby, potentially contribute towards tumor initiation and progression. (see reviews [12,24,82]). 

## 4. mRNA Translation Stress and Feedback to Ribosome Biogenesis

The status of global protein synthesis in proliferating cells depends on the availability of functional ribosomes. Cells use a dynamic process called ribosome homeostasis to strike the balance between the availability of ribosomes in the cytoplasm and the demand of functional ribosomes for mRNA translation. At any state of a cell, the mRNA translation draws ribosomes from its available pool, resulting in the disruption of ribosome homeostasis [77]. After the termination of mRNA translation, the eukaryotic releasing factors (eRF1 and eRF3) act together with the adenosine triphosphate-binding cassette family E member (ABCE1) to separate the 40S and 60S ribosomes and recycle back into the cytoplasm [95,96]. Dysfunctional translation due to ribosome stalling, strong mRNA structure, truncated mRNAs, or a shortage of tRNA supply tend to disturb the ribosome homeostasis greatly. Cells use the surveillance mechanism to retrieve these stalled ribosomes and coordinate with the ribosome recycling machinery in order to restore the ribosome homeostasis [97,98,99,100,101,102] (see review [77]). Interestingly, how mRNA translation stress feeds back to the ribosome biogenesis pathway and how this pathway is synchronized with cell cycle proliferation remains relatively unknown. A recent report has offered an explanation to begin shedding light on this question. 

The system used to study this phenomenon was based on the Epstein–Barr virusnuclear antigen 1 (EBNA1) that harbors oncogenic activity and is essential for viral replication and survival. EBNA1 carries a glycine–alanine repeat sequence (GAr), which spans over 200 residues depending on the viral strain. It turns out this repeat serves two functions for the virus via the same mechanism. The first is that by suppressing its own mRNA translation *in cis* it minimizes the production of EBNA1-derived antigenic peptides for the major histocompatibility (MHC) class I pathway and, thus, helps the virus to evade the immune system [103,104,105]. The translation inhibitory activity of the GAr *in cis* also makes it a unique tool for studying the cellular response to mRNA translation stress, bypassing the use of general protein synthesis inhibitors. This capacity made it possible to demonstrate that GAr-mediated suppression of translation *in cis* also results in an increase in cell proliferation and ribosome biogenesis. By manipulating the 5′ end of EBNA1 constructs, it is possible to override translation suppression and express high levels of the encoded GAr peptide. This results in a loss of cell proliferation, demonstrating that it is not the encoded peptide that affects cell proliferation but the capacity to suppress its own synthesis [55]. 

**Figure 2:**
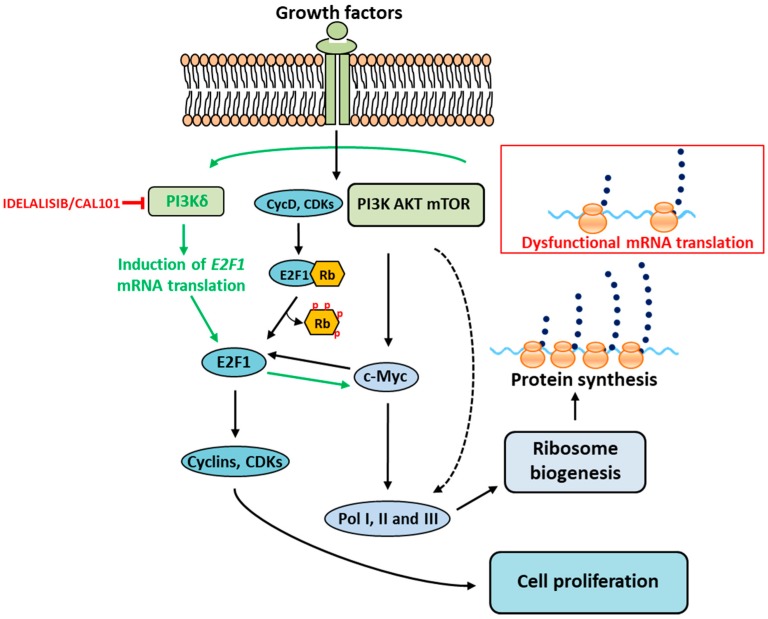
mRNA translation stress feedback to ribosome biogenesis and cell proliferation. Ribosome biogenesis and mRNA translation are large metabolic processes promoting the cellular growth and proliferation. Growth factors trigger the signaling cascades which induce RiBi via c-Myc and cell proliferation by activating the CDKs, E2F1, and cyclins (indicated with black arrows). In the case of dysfunctional mRNA translation (red box), stress signals from polysomes activate PI3Kδ and trigger a novel signaling pathway (indicated with green arrows) independent of AKT and mTOR, resulting in increased translation of *E2F1* mRNA. The newly synthesized active E2F1, in turn, stimulates the downstream transcription targets c-Myc and cyclins to induce ribosome biogenesis and cell proliferation. Idelalisib/CAL-101 is a selective inhibitor of PI3Kδ (indicated with red T-bar). This novel signaling pathway provides the essential cellular feedback response to mRNA translation stress and its synchronization with ribosome biogenesis and cell proliferation.

The induction of cell proliferation was linked with an induction of c-Myc levels and required the E2F1 binding site in the c-Myc promoter. DNA-ChIP assays also showed that the E2F1 binding sequence is critical for c-Myc activation. This pointed to an E2F1-dependent mechanism of GAr-mediated cell proliferation and was supported by the observation that other E2F1 target genes such as cyclins, were also induced and that the effect of the GAr could be reversed by overexpressing the retinoblastoma protein (pRb). Interestingly, the increase in E2F1 protein levels was observed without a change in E2F1 mRNA levels. Overexpression of E1A that binds the pocket of pRb and competes with E2Fs had only a limited stimulatory effect, underlining that the effect of the GAr is not related to preventing the pRb from binding E2F1. This shows that EBNA1 is targeting the E2F1 pathway but unlike other viral oncogenes such as large T, E1A, or E7, it does not affect the pRb–E2F1 interaction. Instead, the explanation for EBNA1-mediated induction of E2F1 expression is through a unique induction of E2F1 mRNA translation. It would be expected that disruption in the translation of one mRNA that leads to induced translation of another would require a signaling pathway. Extensive works were carried out to identify the responsible signaling pathway and, surprisingly, it was shown that the PI3Kδ, specifically, is essential [55]. PI3Kδ belongs to the class IA group of the PI3k family, along with PI3Kα and PI3Kβ [106]. PI3Kδ is predominantly expressed in leukocytes but is also present in tumor cells of solid origin [107,108]. This kinase plays an essential role in inflammatory and allergic responses; both loss and gain of function mutations of PI3Kδ were detected in patients with primary immunodeficiency [109,110]. The participation of PI3Kδ in the mRNA stress response pathway is intriguing, as this kinase is normally associated with a signal cascade of extracellular growth stimuli. However, PI3Kδ is reported to have a gain in function mutations in activated PI3Kδ syndrome (APDS) which is often associated with Epstein–Barr virus (EBV) and other Herpes viral infections [55,106,111]. Also, in the case of Burkitt lymphomagenesis, the PI3K signaling pathway was reported to have oncogenic cooperation with Myc in stimulating the rRNA transcription and ribosome biogenesis [112,113]. More importantly, this is not mediated via the classic RTK-PI3K-AKT pathway and inhibitors of AKT, mTOR, or PI3Kα/β have no effect on E2F1 synthesis. The AKT pathway has a global effect on mRNA translation, but in this case the effect is specific for the E2F1 message, see Figure 2. An inhibitor of PI3Kδ exists in the form of CAL-101 that is used in the clinic (Idelalisib) to treat chronic lymphocytic leukemia, follicular B cell Hodgkin lymphoma, and relapsed small lymphocytic lymphoma [114]. The treatment of cells with CAL-101 prevents GAr-mediated induction of E2F1 and, interestingly, it also suppresses E2F1 levels in non-EBNA1 expressing tumor cell lines. This shows that this translation stress pathway is not specific for EBNA1 but is a general pathway for linking ribosome function with cellular growth and proliferation. Therefore, it is possible that some of the clinical effects of PI3Kδ-inhibitors are mediated via the suppression of E2F1. EBNA1 transgenic mice develop lymphoma, and a reverse phenotype between EBNA1 expression in animals and lymphoma incidence was reported [115,116]. It has been difficult to explain how less of an oncogene can give more cancer but, in fact, it turned out that it is not the EBNA1 protein that is oncogenic—it is the EBNA1 mRNA. Treating these lymphomas with CAL-101 suppressed the expression of Myc and E2F1 and killed the tumor cells [55].

## 5. Conclusions

Overall, this study described a novel oncogenic pathway triggered by PI3Kδ in response to mRNA translation stress, acting via E2F1 and c-Myc and resulting in the induction of ribosome biogenesis and cell proliferation. It also showed that the Epstein–Barr virus encoded EBNA1, like other oncogenic viral factors such as HPV E7, Adeno E1A, or SV40 Large T targets E2F1. However, EBNA1 uses a different mechanism of action that does not interfere with E2F1 binding to pocket proteins such as pRb. This helped explain the oncogenic role of EBV. Also, it demonstrated how a virus in its latent state exploited the ribosomal feedback pathway in order to favor their propagation and oncogenesis. This model could be applied to investigate the oncogenic activity of other latent viruses. 

## References

[1] Schmeing T.M., Ramakrishnan V. (2009). What recent ribosome structures have revealed about the mechanism of translation. Nature.

[2] Armache J.P., Jarasch A., Anger A.M., Villa E., Becker T., Bhushan S., Jossinet F., Habeck M., Dindar G., Franckenberg S. (2010). Cryo-EM structure and rRNA model of a translating eukaryotic 80S ribosome at 5.5-A resolution. Proc. Natl. Acad. Sci. USA.

[3] Melnikov S., Ben-Shem A., Garreau de Loubresse N., Jenner L., Yusupova G., Yusupov M. (2012). One core, two shells: Bacterial and eukaryotic ribosomes. Nat. Struct. Mol. Biol..

[4] Ben-Shem A., Garreau de Loubresse N., Melnikov S., Jenner L., Yusupova G., Yusupov M. (2011). The structure of the eukaryotic ribosome at 3.0 A resolution. Science.

[5] Rabl J., Leibundgut M., Ataide S.F., Haag A., Ban N. (2011). Crystal structure of the eukaryotic 40S ribosomal subunit in complex with initiation factor 1. Science.

[6] Klinge S., Voigts-Hoffmann F., Leibundgut M., Arpagaus S., Ban N. (2011). Crystal structure of the eukaryotic 60S ribosomal subunit in complex with initiation factor 6. Science.

[7] Yusupova G., Yusupov M. (2014). High-resolution structure of the eukaryotic 80S ribosome. Annu. Rev. Biochem..

[8] Chandramouli P., Topf M., Menetret J.F., Eswar N., Cannone J.J., Gutell R.R., Sali A., Akey C.W. (2008). Structure of the mammalian 80S ribosome at 8.7 A resolution. Structure.

[9] Woolford J.L., Baserga S.J. (2013). Ribosome biogenesis in the yeast Saccharomyces cerevisiae. Genetics.

[10] Natchiar S.K., Myasnikov A.G., Kratzat H., Hazemann I., Klaholz B.P. (2017). Visualization of chemical modifications in the human 80S ribosome structure. Nature.

[11] Natchiar S.K., Myasnikov A.G., Hazemann I., Klaholz B.P. (2018). Visualizing the Role of 2’-OH rRNA Methylations in the Human Ribosome Structure. Biomolecules.

[12] Pelletier J., Thomas G., Volarevic S. (2018). Ribosome biogenesis in cancer: New players and therapeutic avenues. Nat. Rev. Cancer.

[13] Gentilella A., Kozma S.C., Thomas G. (2015). A liaison between mTOR signaling, ribosome biogenesis and cancer. Biochim. Biophys. Acta.

[14] Tschochner H., Hurt E. (2003). Pre-ribosomes on the road from the nucleolus to the cytoplasm. Trends Cell Biol..

[15] Ogle J.M., Ramakrishnan V. (2005). Structural insights into translational fidelity. Annu. Rev. Biochem..

[16] Zaher H.S., Green R. (2009). Fidelity at the molecular level: Lessons from protein synthesis. Cell.

[17] Tafforeau L., Zorbas C., Langhendries J.L., Mullineux S.T., Stamatopoulou V., Mullier R., Wacheul L., Lafontaine D.L. (2013). The complexity of human ribosome biogenesis revealed by systematic nucleolar screening of Pre-rRNA processing factors. Mol. Cell.

[18] Wild T., Horvath P., Wyler E., Widmann B., Badertscher L., Zemp I., Kozak K., Csucs G., Lund E., Kutay U. (2010). A protein inventory of human ribosome biogenesis reveals an essential function of exportin 5 in 60S subunit export. PLoS Biol..

[19] Badertscher L., Wild T., Montellese C., Alexander L.T., Bammert L., Sarazova M., Stebler M., Csucs G., Mayer T.U., Zamboni N. (2015). Genome-wide RNAi Screening Identifies Protein Modules Required for 40S Subunit Synthesis in Human Cells. Cell Rep..

[20] O’Donohue M.F., Choesmel V., Faubladier M., Fichant G., Gleizes P.E. (2010). Functional dichotomy of ribosomal proteins during the synthesis of mammalian 40S ribosomal subunits. J. Cell Biol..

[21] Farley K.I., Baserga S.J. (2016). Probing the mechanisms underlying human diseases in making ribosomes. Biochem. Soc. Trans..

[22] Grummt I. (2003). Life on a planet of its own: Regulation of RNA polymerase I transcription in the nucleolus. Genes Dev..

[23] Bodem J., Dobreva G., Hoffmann-Rohrer U., Iben S., Zentgraf H., Delius H., Vingron M., Grummt I. (2000). TIF-IA, the factor mediating growth-dependent control of ribosomal RNA synthesis, is the mammalian homolog of yeast Rrn3p. EMBO Rep..

[24] Derenzini M., Montanaro L., Trere D. (2017). Ribosome biogenesis and cancer. Acta Histochem..

[25] Brighenti E., Trere D., Derenzini M. (2015). Targeted cancer therapy with ribosome biogenesis inhibitors: A real possibility?. Oncotarget.

[26] Stepanchick A., Zhi H., Cavanaugh A.H., Rothblum K., Schneider D.A., Rothblum L.I. (2013). DNA binding by the ribosomal DNA transcription factor rrn3 is essential for ribosomal DNA transcription. J. Biol. Chem..

[27] Kressler D., Hurt E., Bassler J. (2010). Driving ribosome assembly. Biochim. Biophys. Acta.

[28] Grummt I. (2010). Wisely chosen paths–regulation of rRNA synthesis: Delivered on 30 June 2010 at the 35th FEBS Congress in Gothenburg, Sweden. FEBS J..

[29] de la Cruz J., Karbstein K., Woolford J.L. (2015). Functions of ribosomal proteins in assembly of eukaryotic ribosomes in vivo. Annu. Rev. Biochem..

[30] Thomas G. (2000). An encore for ribosome biogenesis in the control of cell proliferation. Nat. Cell Biol..

[31] Volarevic S., Stewart M.J., Ledermann B., Zilberman F., Terracciano L., Montini E., Grompe M., Kozma S.C., Thomas G. (2000). Proliferation, but not growth, blocked by conditional deletion of 40S ribosomal protein S6. Science.

[32] Teng T., Thomas G., Mercer C.A. (2013). Growth control and ribosomopathies. Curr. Opin. Genet. Dev..

[33] Stefanovsky V.Y., Pelletier G., Hannan R., Gagnon-Kugler T., Rothblum L.I., Moss T. (2001). An immediate response of ribosomal transcription to growth factor stimulation in mammals is mediated by ERK phosphorylation of UBF. Mol. Cell.

[34] Stefanovsky V., Langlois F., Gagnon-Kugler T., Rothblum L.I., Moss T. (2006). Growth factor signaling regulates elongation of RNA polymerase I transcription in mammals via UBF phosphorylation and r-chromatin remodeling. Mol. Cell.

[35] Plas D.R., Thomas G. (2009). Tubers and tumors: Rapamycin therapy for benign and malignant tumors. Curr. Opin. Cell Biol..

[36] Mayer C., Zhao J., Yuan X., Grummt I. (2004). mTOR-dependent activation of the transcription factor TIF-IA links rRNA synthesis to nutrient availability. Genes Dev..

[37] Hannan K.M., Brandenburger Y., Jenkins A., Sharkey K., Cavanaugh A., Rothblum L., Moss T., Poortinga G., McArthur G.A., Pearson R.B. (2003). mTOR-dependent regulation of ribosomal gene transcription requires S6K1 and is mediated by phosphorylation of the carboxy-terminal activation domain of the nucleolar transcription factor UBF. Mol. Cell. Biol..

[38] Kantidakis T., Ramsbottom B.A., Birch J.L., Dowding S.N., White R.J. (2010). mTOR associates with TFIIIC, is found at tRNA and 5S rRNA genes, and targets their repressor Maf1. Proc. Natl. Acad. Sci. USA.

[39] Mayer C., Grummt I. (2006). Ribosome biogenesis and cell growth: MTOR coordinates transcription by all three classes of nuclear RNA polymerases. Oncogene.

[40] Iadevaia V., Liu R., Proud C.G. (2014). mTORC1 signaling controls multiple steps in ribosome biogenesis. Sem. Cell Dev. Biol..

[41] Zinzalla V., Stracka D., Oppliger W., Hall M.N. (2011). Activation of mTORC2 by association with the ribosome. Cell.

[42] Zhu J., Blenis J., Yuan J. (2008). Activation of PI3K/Akt and MAPK pathways regulates Myc-mediated transcription by phosphorylating and promoting the degradation of Mad1. Proc. Natl. Acad. Sci. USA.

[43] Gomez-Roman N., Felton-Edkins Z.A., Kenneth N.S., Goodfellow S.J., Athineos D., Zhang J., Ramsbottom B.A., Innes F., Kantidakis T., Kerr E.R. (2006). Activation by c-Myc of transcription by RNA polymerases I, II and III. Biochem. Soc. Symp..

[44] van Riggelen J., Yetil A., Felsher D.W. (2010). MYC as a regulator of ribosome biogenesis and protein synthesis. Nat. Rev. Cancer.

[45] Sirri V., Roussel P., Hernandez-Verdun D. (2000). In vivo release of mitotic silencing of ribosomal gene transcription does not give rise to precursor ribosomal RNA processing. J. Cell Biol..

[46] Heix J., Vente A., Voit R., Budde A., Michaelidis T.M., Grummt I. (1998). Mitotic silencing of human rRNA synthesis: Inactivation of the promoter selectivity factor SL1 by cdc2/cyclin B-mediated phosphorylation. EMBO J..

[47] Sirri V., Roussel P., Hernandez-Verdun D. (1999). The mitotically phosphorylated form of the transcription termination factor TTF-1 is associated with the repressed rDNA transcription machinery. J. Cell Sci..

[48] Voit R., Seiler J., Grummt I. (2015). Cooperative Action of Cdk1/cyclin B and SIRT1 Is Required for Mitotic Repression of rRNA Synthesis. PLoS Genet..

[49] Cavanaugh A.H., Hempel W.M., Taylor L.J., Rogalsky V., Todorov G., Rothblum L.I. (1995). Activity of RNA polymerase I transcription factor UBF blocked by Rb gene product. Nature.

[50] Voit R., Schafer K., Grummt I. (1997). Mechanism of repression of RNA polymerase I transcription by the retinoblastoma protein. Mol. Cell. Biol..

[51] Hannan K.M., Hannan R.D., Smith S.D., Jefferson L.S., Lun M., Rothblum L.I. (2000). Rb and p130 regulate RNA polymerase I transcription: Rb disrupts the interaction between UBF and SL-1. Oncogene.

[52] Ciarmatori S., Scott P.H., Sutcliffe J.E., McLees A., Alzuherri H.M., Dannenberg J.H., te Riele H., Grummt I., Voit R., White R.J. (2001). Overlapping functions of the pRb family in the regulation of rRNA synthesis. Mol. Cell. Biol..

[53] White R.J., Trouche D., Martin K., Jackson S.P., Kouzarides T. (1996). Repression of RNA polymerase III transcription by the retinoblastoma protein. Nature.

[54] Felton-Edkins Z.A., Kenneth N.S., Brown T.R., Daly N.L., Gomez-Roman N., Grandori C., Eisenman R.N., White R.J. (2003). Direct regulation of RNA polymerase III transcription by RB, p53 and c-Myc. Cell Cycle.

[55] Gnanasundram S.V., Pyndiah S., Daskalogianni C., Armfield K., Nylander K., Wilson J.B., Fahraeus R. (2017). PI3Kδ activates E2F1 synthesis in response to mRNA translation stress. Nat. Commun..

[56] Harbour J.W., Luo R.X., Dei Santi A., Postigo A.A., Dean D.C. (1999). Cdk phosphorylation triggers sequential intramolecular interactions that progressively block Rb functions as cells move through G1. Cell.

[57] Chen H.Z., Tsai S.Y., Leone G. (2009). Emerging roles of E2Fs in cancer: An exit from cell cycle control. Nat. Rev. Cancer.

[58] Whyte P., Buchkovich K.J., Horowitz J.M., Friend S.H., Raybuck M., Weinberg R.A., Harlow E. (1988). Association between an oncogene and an anti-oncogene: The adenovirus E1A proteins bind to the retinoblastoma gene product. Nature.

[59] Phelps W.C., Yee C.L., Munger K., Howley P.M. (1988). The human papillomavirus type 16 E7 gene encodes transactivation and transformation functions similar to those of adenovirus E1A. Cell.

[60] Felsani A., Mileo A.M., Paggi M.G. (2006). Retinoblastoma family proteins as key targets of the small DNA virus oncoproteins. Oncogene.

[61] Sherr C.J., Roberts J.M. (1999). CDK inhibitors: Positive and negative regulators of G1-phase progression. Genes Dev..

[62] David-Pfeuty T. (2006). The flexible evolutionary anchorage-dependent Pardee’s restriction point of mammalian cells: How its deregulation may lead to cancer. Biochim. Biophys. Acta.

[63] Donati G., Peddigari S., Mercer C.A., Thomas G. (2013). 5S ribosomal RNA is an essential component of a nascent ribosomal precursor complex that regulates the Hdm2-p53 checkpoint. Cell Rep..

[64] Bursac S., Brdovcak M.C., Donati G., Volarevic S. (2014). Activation of the tumor suppressor p53 upon impairment of ribosome biogenesis. Biochim. Biophys. Acta.

[65] Deisenroth C., Zhang Y. (2010). Ribosome biogenesis surveillance: Probing the ribosomal protein-Mdm2-p53 pathway. Oncogene.

[66] Opferman J.T., Zambetti G.P. (2006). Translational research? Ribosome integrity and a new p53 tumor suppressor checkpoint. Cell Death Differ..

[67] Zhang Y., Lu H. (2009). Signaling to p53: Ribosomal proteins find their way. Cancer Cell.

[68] Mayer C., Grummt I. (2005). Cellular stress and nucleolar function. Cell Cycle.

[69] Draptchinskaia N., Gustavsson P., Andersson B., Pettersson M., Willig T.N., Dianzani I., Ball S., Tchernia G., Klar J., Matsson H. (1999). The gene encoding ribosomal protein S19 is mutated in Diamond-Blackfan anaemia. Nat. Genet..

[70] Ebert B.L., Pretz J., Bosco J., Chang C.Y., Tamayo P., Galili N., Raza A., Root D.E., Attar E., Ellis S.R. (2008). Identification of RPS14 as a 5q− syndrome gene by RNA interference screen. Nature.

[71] Farrar J.E., Vlachos A., Atsidaftos E., Carlson-Donohoe H., Markello T.C., Arceci R.J., Ellis S.R., Lipton J.M., Bodine D.M. (2011). Ribosomal protein gene deletions in Diamond-Blackfan anemia. Blood.

[72] Shwachman H., Diamond L.K., Oski F.A., Khaw K.T. (1964). The Syndrome of Pancreatic Insufficiency and Bone Marrow Dysfunction. J. Pediatr..

[73] Bolze A., Mahlaoui N., Byun M., Turner B., Trede N., Ellis S.R., Abhyankar A., Itan Y., Patin E., Brebner S. (2013). Ribosomal protein SA haploinsufficiency in humans with isolated congenital asplenia. Science.

[74] Danilova N., Gazda H.T. (2015). Ribosomopathies: How a common root can cause a tree of pathologies. Dis. Models Mech..

[75] Narla A., Ebert B.L. (2010). Ribosomopathies: Human disorders of ribosome dysfunction. Blood.

[76] Tahmasebi S., Khoutorsky A., Mathews M.B., Sonenberg N. (2018). Translation deregulation in human disease. Nat. Rev. Mol. Cell Biol..

[77] Mills E.W., Green R. (2017). Ribosomopathies: There’s strength in numbers. Science.

[78] Xue S., Barna M. (2012). Specialized ribosomes: A new frontier in gene regulation and organismal biology. Nat. Rev. Mol. Cell Biol..

[79] Genuth N.R., Barna M. (2018). Heterogeneity and specialized functions of translation machinery: From genes to organisms. Nat. Rev. Genet..

[80] Shi Z., Fujii K., Kovary K.M., Genuth N.R., Rost H.L., Teruel M.N., Barna M. (2017). Heterogeneous Ribosomes Preferentially Translate Distinct Subpools of mRNAs Genome-wide. Mol. Cell.

[81] Genuth N.R., Barna M. (2018). The Discovery of Ribosome Heterogeneity and Its Implications for Gene Regulation and Organismal Life. Mol. Cell.

[82] Bustelo X.R., Dosil M. (2018). Ribosome biogenesis and cancer: Basic and translational challenges. Curr. Opin. Genet. Dev..

[83] Barna M., Pusic A., Zollo O., Costa M., Kondrashov N., Rego E., Rao P.H., Ruggero D. (2008). Suppression of Myc oncogenic activity by ribosomal protein haploinsufficiency. Nature.

[84] Rossetti S., Hoogeveen A.T., Esposito J., Sacchi N. (2010). Loss of MTG16a (CBFA2T3), a novel rDNA repressor, leads to increased ribogenesis and disruption of breast acinar morphogenesis. J. Cell. Mol. Med..

[85] Brighenti E., Calabrese C., Liguori G., Giannone F.A., Trere D., Montanaro L., Derenzini M. (2014). Interleukin 6 downregulates p53 expression and activity by stimulating ribosome biogenesis: A new pathway connecting inflammation to cancer. Oncogene.

[86] Donati G., Bertoni S., Brighenti E., Vici M., Trere D., Volarevic S., Montanaro L., Derenzini M. (2011). The balance between rRNA and ribosomal protein synthesis up- and downregulates the tumour suppressor p53 in mammalian cells. Oncogene.

[87] Erales J., Marchand V., Panthu B., Gillot S., Belin S., Ghayad S.E., Garcia M., Laforets F., Marcel V., Baudin-Baillieu A. (2017). Evidence for rRNA 2′-O-methylation plasticity: Control of intrinsic translational capabilities of human ribosomes. Proc. Natl. Acad. Sci. USA.

[88] Marcel V., Ghayad S.E., Belin S., Therizols G., Morel A.P., Solano-Gonzalez E., Vendrell J.A., Hacot S., Mertani H.C., Albaret M.A. (2013). p53 acts as a safeguard of translational control by regulating fibrillarin and rRNA methylation in cancer. Cancer Cell.

[89] Marcel V., Catez F., Diaz J.J. (2015). Ribosome heterogeneity in tumorigenesis: The rRNA point of view. Mol. Cell. Oncol..

[90] Monaco P.L., Marcel V., Diaz J.J., Catez F. (2018). 2’-*O*-Methylation of Ribosomal RNA: Towards an Epitranscriptomic Control of Translation?. Biomolecules.

[91] Belin S., Beghin A., Solano-Gonzalez E., Bezin L., Brunet-Manquat S., Textoris J., Prats A.C., Mertani H.C., Dumontet C., Diaz J.J. (2009). Dysregulation of ribosome biogenesis and translational capacity is associated with tumor progression of human breast cancer cells. PLoS ONE.

[92] Rocchi L., Pacilli A., Sethi R., Penzo M., Schneider R.J., Trere D., Brigotti M., Montanaro L. (2013). Dyskerin depletion increases VEGF mRNA internal ribosome entry site-mediated translation. Nucleic Acids Res..

[93] Yoon A., Peng G., Brandenburger Y., Zollo O., Xu W., Rego E., Ruggero D. (2006). Impaired control of IRES-mediated translation in X-linked dyskeratosis congenita. Science.

[94] Penzo M., Montanaro L. (2018). Turning Uridines around: Role of rRNA Pseudouridylation in Ribosome Biogenesis and Ribosomal Function. Biomolecules.

[95] Shoemaker C.J., Green R. (2011). Kinetic analysis reveals the ordered coupling of translation termination and ribosome recycling in yeast. Proc. Natl. Acad. Sci. USA.

[96] Pisarev A.V., Skabkin M.A., Pisareva V.P., Skabkina O.V., Rakotondrafara A.M., Hentze M.W., Hellen C.U., Pestova T.V. (2010). The role of ABCE1 in eukaryotic posttermination ribosomal recycling. Mol. Cell.

[97] Shah P., Ding Y., Niemczyk M., Kudla G., Plotkin J.B. (2013). Rate-limiting steps in yeast protein translation. Cell.

[98] Tenson T., Ehrenberg M. (2002). Regulatory nascent peptides in the ribosomal tunnel. Cell.

[99] Wen J.D., Lancaster L., Hodges C., Zeri A.C., Yoshimura S.H., Noller H.F., Bustamante C., Tinoco I. (2008). Following translation by single ribosomes one codon at a time. Nature.

[100] Simms C.L., Hudson B.H., Mosior J.W., Rangwala A.S., Zaher H.S. (2014). An active role for the ribosome in determining the fate of oxidized mRNA. Cell Rep..

[101] Guydosh N.R., Green R. (2014). Dom34 rescues ribosomes in 3′ untranslated regions. Cell.

[102] Ishimura R., Nagy G., Dotu I., Zhou H., Yang X.L., Schimmel P., Senju S., Nishimura Y., Chuang J.H., Ackerman S.L. (2014). RNA function. Ribosome stalling induced by mutation of a CNS-specific tRNA causes neurodegeneration. Science.

[103] Yin Y., Manoury B., Fahraeus R. (2003). Self-inhibition of synthesis and antigen presentation by Epstein-Barr virus-encoded EBNA1. Science.

[104] Apcher S., Daskalogianni C., Manoury B., Fahraeus R. (2010). Epstein Barr virus-encoded EBNA1 interference with MHC class I antigen presentation reveals a close correlation between mRNA translation initiation and antigen presentation. PLoS Pathog..

[105] Murat P., Zhong J., Lekieffre L., Cowieson N.P., Clancy J.L., Preiss T., Balasubramanian S., Khanna R., Tellam J. (2014). G-quadruplexes regulate Epstein-Barr virus-encoded nuclear antigen 1 mRNA translation. Nat. Chem. Biol..

[106] Thorpe L.M., Yuzugullu H., Zhao J.J. (2015). PI3K in cancer: Divergent roles of isoforms, modes of activation and therapeutic targeting. Nat. Rev. Cancer.

[107] Okkenhaug K. (2013). Signaling by the phosphoinositide 3-kinase family in immune cells. Annu. Rev. Immunol..

[108] Sawyer C., Sturge J., Bennett D.C., O’Hare M.J., Allen W.E., Bain J., Jones G.E., Vanhaesebroeck B. (2003). Regulation of breast cancer cell chemotaxis by the phosphoinositide 3-kinase p110δ. Cancer Res..

[109] Koyasu S. (2003). The role of PI3K in immune cells. Nat. Immunol..

[110] Ali K., Bilancio A., Thomas M., Pearce W., Gilfillan A.M., Tkaczyk C., Kuehn N., Gray A., Giddings J., Peskett E. (2004). Essential role for the p110δ phosphoinositide 3-kinase in the allergic response. Nature.

[111] Lucas C.L., Chandra A., Nejentsev S., Condliffe A.M., Okkenhaug K. (2016). PI3Kδ and primary immunodeficiencies. Nat. Rev. Immunol..

[112] Derenzini E., Rossi A., Trere D. (2018). Treating hematological malignancies with drugs inhibiting ribosome biogenesis: When and why. J. Hematol. Oncol..

[113] Sander S., Calado D.P., Srinivasan L., Kochert K., Zhang B., Rosolowski M., Rodig S.J., Holzmann K., Stilgenbauer S., Siebert R. (2012). Synergy between PI3K signaling and MYC in Burkitt lymphomagenesis. Cancer Cell.

[114] Furman R.R., Sharman J.P., Coutre S.E., Cheson B.D., Pagel J.M., Hillmen P., Barrientos J.C., Zelenetz A.D., Kipps T.J., Flinn I. (2014). Idelalisib and rituximab in relapsed chronic lymphocytic leukemia. N. Engl. J. Med..

[115] Wilson J.B., Bell J.L., Levine A.J. (1996). Expression of Epstein-Barr virus nuclear antigen-1 induces B cell neoplasia in transgenic mice. EMBO J..

[116] Kang M.S., Lu H., Yasui T., Sharpe A., Warren H., Cahir-McFarland E., Bronson R., Hung S.C., Kieff E. (2005). Epstein-Barr virus nuclear antigen 1 does not induce lymphoma in transgenic FVB mice. Proc. Natl. Acad. Sci. USA.

